# Motor Imagery Training of Reaching-to-Grasp Movement Supplemented by a Virtual Environment in an Individual With Congenital Bilateral Transverse Upper-Limb Deficiency

**DOI:** 10.3389/fpsyg.2021.638780

**Published:** 2021-03-22

**Authors:** Joanna Mencel, Anna Jaskólska, Jarosław Marusiak, Łukasz Kamiński, Marek Kurzyński, Andrzej Wołczowski, Artur Jaskólski, Katarzyna Kisiel-Sajewicz

**Affiliations:** ^1^Department of Kinesiology, Faculty of Physiotherapy, University School of Physical Education in Wrocław, Wrocław, Poland; ^2^Department of Systems and Computer Networks, Faculty of Electronics, Wrocław University of Science and Technology, Wrocław, Poland; ^3^Department of Fundamental Cybernetics and Robotics, Institute of Computer Engineering, Control and Robotics, Wrocław University of Science and Technology, Wrocław, Poland

**Keywords:** mental training, reaching, grasping, EEG, amelia, neuroplasticity, transplantation

## Abstract

This study explored the effect of kinesthetic motor imagery training on reaching-to-grasp movement supplemented by a virtual environment in a patient with congenital bilateral transverse upper-limb deficiency. Based on a theoretical assumption, it is possible to conduct such training in this patient. The aim of this study was to evaluate whether cortical activity related to motor imagery of reaching and motor imagery of grasping of the right upper limb was changed by computer-aided imagery training (CAIT) in a patient who was born without upper limbs compared to a healthy control subject, as characterized by multi-channel electroencephalography (EEG) signals recorded before and 4, 8, and 12 weeks after CAIT. The main task during CAIT was to kinesthetically imagine the execution of reaching-to-grasp movements without any muscle activation, supplemented by computer visualization of movements provided by a special headset. Our experiment showed that CAIT can be conducted in the patient with higher vividness of imagery for reaching than grasping tasks. Our results confirm that CAIT can change brain activation patterns in areas related to motor planning and the execution of reaching and grasping movements, and that the effect was more pronounced in the patient than in the healthy control subject. The results show that CAIT has a different effect on the cortical activity related to the motor imagery of a reaching task than on the cortical activity related to the motor imagery of a grasping task. The change observed in the activation patterns could indicate CAIT-induced neuroplasticity, which could potentially be useful in rehabilitation or brain-computer interface purposes for such patients, especially before and after transplantation. This study was part of a registered experiment (ID: NCT04048083).

## Introduction

Motor imagery is defined as the conscious, mental simulation of an action without any body movement (Jeannerod, 1994; Jeannerod and Decety, 1995). Imaging studies have shown that the motor imagery of movement and an execution of movements share overlapping brain areas including the primary motor cortex, supplementary motor area, superior and inferior parietal lobules, dorsal and ventral premotor cortices, prefrontal cortex, inferior frontal gyrus, superior temporal gyrus, sensory cortex, anterior cingulate gyrus, basal ganglia, and cerebellum (Decety et al., 1994; Jeannerod and Decety, 1995; Stephan et al., 1995; Fadiga et al., 1999; Gerardin et al., 2000; Hanakawa et al., 2003; Solodkin et al., 2004; Avanzino et al., 2015). There are two main types of motor imagery: kinesthetic, which can be defined as mentally feeling oneself moving one’s own body parts, and visual, which can be described as mentally seeing another person moving his/her body parts. Most authors agree that the kinesthetic approach to imagery is more effective and produces higher levels of physiological responses (Ranganathan et al., 2004; Harris and Hebert, 2015). Motor imagery plays an important role in motor skill learning (Mokienko et al., 2013; Cabral-Sequeira et al., 2016; Sobierajewicz et al., 2016) and rehabilitation (Mulder, 2007; Ietswaart et al., 2011; Harris and Hebert, 2015). It is also used in the brain-computer interface field (Ortner et al., 2012; Mokienko et al., 2013; Teo and Chew, 2014).

The effectiveness of motor imagery is related to training-induced neuroplasticity, as the brain has the capacity to adapt to both internal and external stimuli (Merzenich et al., 2014; Marins et al., 2019). Mental training, as well as physical training, is a stimulus that can induce activity-dependent neuroplasticity (Di Rienzo et al., 2014; Ballesteros et al., 2018). On one hand, motor imagery is known to be a useful approach for rehabilitation in patients with altered body image such as those with spinal cord injuries (Saadah and Melzack, 1994) or those with peripheral lesions, including amputees (Ersland et al., 1996; Lotze et al., 2001). The efficacy of motor imagery in reducing phantom limb pain has also been assessed (Harris and Hebert, 2015). On the other hand, there have been cases of people with congenital limb deficiencies and altered body image (Melzack et al., 1997; Gallagher et al., 1998; Brugger et al., 2000), whose phantom sensations of nonexistent body parts have been reported by many authors (Melzack et al., 1997; Brugger et al., 2000), whereas this phenomenon was disproved by the study of others (Montoya et al., 1997; Flor et al., 1998; Reilly and Sirigu, 2011). Montoya et al. (1997) showed that the homuncular organization of the somatosensory cortex in subjects with congenital limb atrophy is similar to that in healthy subjects. There were also no significant differences between the locations of the cortical maps and the distances between the hand digits, lower lip, and toes in such patients compared to healthy controls. Flor et al. (1998) confirmed no reorganization of the lower lip map in the primary somatosensory cortex of subjects with congenital limb deficiency and further suggested the presence of a silent representation of the missing limb in such patients. Additionally, it has been shown that the neural representation of tools and hands appears to be virtually the same in individuals born without hands as in healthy people (Striem-Amit et al., 2017), as well as the spatial arrangement and functional properties of brain areas specialized in the processing of observed actions for the hand (Vannuscorps et al., 2018).

Excluding our previous publication (Kurzynski et al., 2017), we did not find any other literature regarding the use of motor imagery training in patients born without upper limbs, nor any information about the use of any kind of training in these patients. Motor imagery recruits high-level brain processes involved in motor behavior and could be one of the few that can be used in these patients. If training results in training-dependent neuroplasticity, it may be possible to transplant the limbs in these patients in the future. Currently, it seems impossible, but it is not the first time that motor imagery training is used in such unusual situations (Guillot and Debarnot, 2019), and many authors agree that it should be conducted in situations, where traditional therapeutic techniques cannot be implemented. Therefore, we evaluated whether kinesthetic motor imagery training of upper-limb movement (specifically, a functional reaching-to-grasp movement) could promote neuroplasticity in the sensorimotor cortex in a congenitally amputated patient, which could be especially important in the context of transplant surgery. We employed a virtual environment in which movements that were imagined during training were visualized to support the training effect after the importance of the role of visual feedback was proven (Ramachandran and Altschuler, 2009). In this paper, we describe cortical activity associated with the motor imagery of reaching (MIR) and the motor imagery of grasping (MIG) by a patient with a congenital bilateral transverse upper-limb deficiency compared with that of a healthy control subject, as characterized by electroencephalography (EEG) signals recorded before computer-aided imagery training (CAIT) and after 4, 8, and 12 weeks of training. In addition to the neurophysiological method of CAIT evaluation (EEG), we decided to apply a subjective assessment of the imageries’ vividness using a 10-cm visual analog scale (VAS) after Guillot and Collet (2005) reported that questionnaires and self-report ratings are reliable psychometric methods for evaluating the quality of motor imagery in healthy subjects. This has never been researched in patients born without upper limbs; however, in healthy people, the spatial patterns of neural activity within the motor cortices assessed by fMRI reflect the individual vividness of imaged tasks (Zabicki et al., 2019).

The aims of this study were (i) to evaluate whether cortical activity related to MIR and MIG of the right upper limb was changed by CAIT in a patient with congenital bilateral transverse upper-limb deficiency and a healthy control subject and (ii) to compare the effects of CAIT on the motor imagery of two different mental tasks: reaching and grasping.

We focused on an analysis of event-related potentials (ERP amplitude, the amplitude of negative potential) after their sensitivity to mental and physical practice was proven (Ranganathan et al., 2004; Allami et al., 2014) and (ii) sources of ERP localization.

## Materials and Methods

### Subjects

The test participant (hereinafter, the patient) was a woman with congenital bilateral transverse upper-limb deficiency who lacked upper limbs at the glenohumeral joint level. The control participant (hereinafter, the control) was a woman with an average body build and no history of neurological, muscular, or skeletal disorders. Both subjects were between 27 and 30 years of age.

The participants were informed about the nature of the experiment, and they provided written consent to participate. The study was approved by the Ethical Committee of the University School of Physical Education in Wroclaw and was conducted in accordance with the Declaration of Helsinki.

### Experimental Procedures

**Figure 1:**
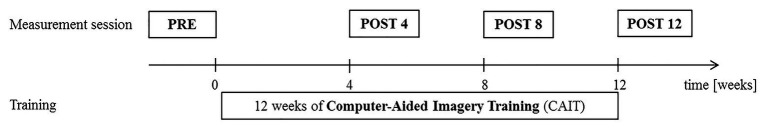
Scheme of the experimental protocol consisting of four measurement sessions (PRE, POST4, POST8, and POST12) and 12 weeks of computer-aided imagery training (CAIT).

The participants received 12 weeks of motor imagery training supplemented by a virtual environment (CAIT). The 12-week training duration was chosen to provoke long-term changes in the nervous system, as suggested by Schuster et al. (2011). Before the training (PRE) and after 4, 8, and 12 weeks of CAIT (POST4, POST8, and POST12, respectively), the subjects participated in the measurement sessions (Figure 1).

### Computer-Aided Imagery Training

The CAIT involved mental training in reaching-to-grasp movements supplemented by the computer visualization of movements provided by a special headset (Sony HMZ-T1 model). The visualization software was coded specifically for this experiment (for details, see Kurzynski et al., 2017). The main task during the CAIT was to kinesthetically imagine the execution of reaching-to-grasp movements without any overt movement or muscle activation (Mizuguchi et al., 2011). We chose the kinesthetic approach for motor imagery training to achieve a physiological response (Ranganathan et al., 2004), and thus potentially provoke neuroplasticity. A virtual reality environment was used for two main reasons: first, to facilitate the motor imagery and make it easier for the patient and, second, to serve as a form of virtual afferent information to create the illusion of possessing a real upper limb. This kind of stimulation could arouse sensations incompatible with a sensory body plan (Guterstam et al., 2011).

During each training session, small vibrating devices were attached to the skin above the trapezius muscle of both subjects and to the first dorsal interosseous muscle of the control to obtain additional sensory information and to elucidate movements. The first vibration was delivered when the subject approached the object to be grasped (a book) with the virtual limb to emphasize the end of the reaching phase, and the second vibration was delivered at the beginning of the grasping phase. The vibration frequency and force were adjusted to the individual subjects’ sensations and remained at the lowest detectable level.

### Training Procedure (CAIT)

During each training session, the subjects were seated in a comfortable chair. The vibrating devices were attached to the skin above the chosen muscles by the same experienced physiotherapist. The patient with only the lower limbs, as well as the healthy control, both touched a real book (always the same book, similar to that presented in the computer animation) to feel the weight of the book, its size, and its surface. The subjects also observed during the CAIT that the location of the book was always the same at approximately 0.14 m away.

While sitting in the experimental chair and wearing a headset, the subject was instructed to relax the body and mind (i.e., to not think about anything in particular). Instruction was then given to perform the mental reaching and precise grasping of the book using the thumb and four fingers with computer animation.

Instructions were given to the subjects at the beginning of each training session, and the subjects performed three practice trials according to the instructions. Instruction was then discontinued, and the subjects performed 30 trials of mental movements, wherein the signal for the start of a trial was equal to the start of the computer animation. Each training session consisted of 30 trials of the task of reaching to grasp (three sets, 10 trials in each set) for the left and right upper limbs separately. There was a 20-s rest between trials, a 3-min rest between sets, and a 15-min rest between sides (right and left upper limb). Each training session took 52 min. A total of 36 training sessions were conducted over 12 weeks (three times a week for 12 weeks).

### Measurement Sessions

All measurements were performed during four sessions, denoted herein as PRE (before CAIT), POST4 (after 4 weeks of CAIT), POST8 (after 8 weeks of CAIT), and POST12 (after 12 weeks of CAIT). Each session consisted of a short introductory period to familiarize the subjects with the tasks to be performed, followed by two motor imagery (MI) tasks: MIR of the right upper limb and MIG of the right hand, with a short break between the two tasks. The subjects were seated comfortably with their eyes open, and their role was to imagine kinesthetically reaching with the right upper limb for a book (the first task) and then to imagine kinesthetically grasping the book (the second task). Each task was repeated 20 times to achieve a sufficient number of trials for further analysis while avoiding fatigue. The protocol for the MI tasks was the same for both tasks. A single tone was the signal to start the imagery, and a double tone was the signal to stop and relax. The subjects did not receive any additional stimuli (visual or tactile stimuli) during the recordings. The time between the single and double tones (i.e., the time for one imagery trial) was 8 s, and the rest time between trials was 10 s. During one trial of the MIR task, the subject was asked to imagine the reaching task upon hearing the single tone and mentally reaching the goal (the book, with its location similar to that used during CAIT) and then to stop the MIR task and wait for another single tone (or an earlier double tone to relax and then another single tone to repeat the MIR task). Each trial of the MIG task lasted exactly 8 s, during which the subject was asked to imagine grasping the book with a force sufficient to lift it upon hearing a single tone and to stop upon hearing a double tone.

During each session (before and after 4, 8, and 12 weeks of training), immediately after EEG data recording, the vividness of the imagination (the ability to create images characterized by a high level of complexity and detail) of the reaching and grasping tasks was evaluated. We used a VAS ranging from 0 = very hard to feel to 10 = very easy to feel.

### EEG Data Recording

EEG data were recorded continuously using Ag/AgCl electrodes from 128 scalp electrodes relative to a vertex reference, using the BioSemi Active-two system (Biosemi Inc., Amsterdam, the Netherlands). The electrodes were placed according to the BioSemi-designed Equiradial system and partially overlapped with an extended International 10–20 system (Oostenveld and Praamstra, 2001) using a nylon electrode cap. The EEG signals were amplified with a bandpass of 0–128 Hz and sampled at 2048 Hz. The impedance between each electrode and the skin was displayed on a computer monitor to inspect the quality of the connection. If a particular electrode showed high impedance, an adjustment was made, such as applying pressure or adding more conducting gel (Electro-gel™, Electro-Cap International Inc., Eaton, OH) to improve the connection. The EEG data recording did not begin until the impedance for all electrodes was below 5 kΩ.

The subjects were instructed to maintain a stable body position and to avoid eye blinking, teeth biting, and head movements during the trials. Minimal eye blinking and body adjustments were allowed during rest periods between trials and tasks. All possible aural and visual sources of distraction were minimized.

### EEG Data Processing and Analysis

The EEG signal data were analyzed using the Brain Electrical Source Analysis 6.1 software (BESA, MEGIS Software GmbH, Gräfelfing, Germany). During offline processing, the EEG signal was downsampled to 512 Hz (Decimator, Biosemi Inc.), visually inspected, and filtered using high (50 Hz) and low (0.53 Hz) filters as well as a notch filter at 50 Hz. Although the subjects were instructed not to perform blinking, biting, or head movements, these activities occasionally occurred, and recordings that included these activities were excluded from further analysis. We calculated the intraclass correlation coefficient (ICC) to determine the intra-rater reliability for the patient and the control for both conditions (two tasks: MIR and MIG), and for four measurement time points (PRE, POST4, POST8, and POST12). We noted poor (less than 0.4; according to Cicchetti, 1994) ICC for the patient and the MIG task, fair ICC for the patient and the MIR task, and excellent ICC for the control (for both tasks). To obtain the ERP, the EEG signals associated with the MIR and MIG tasks for each subject were trigger-averaged using a single tone as the trigger. A 2-s-long time window was chosen for this purpose (where 0 was the time of the single tone and indicated that the subject should start the MI task), with an interval of −100 to 0 ms used for baseline correction. Artifact-free, averaged-referenced trial data were used for further analysis, specifically 16, 14, 15, and 12 trials for the reaching task and 17, 15, 15, and 12 trials over four sessions for the grasping task for the patient (from PRE to POST12); and 19, 20, 15, and 17 trials for the reaching task and 20, 19, 19, and 17 trials over four sessions for the grasping task for the control (from PRE to POST12). Two parameters were calculated to quantify CAIT-induced cortical signal alterations: ERP amplitude and ERP sources.

The ERP amplitudes [the value of the negative potential’s peak, NP (μV)] were obtained from the following regions related to planning and execution of movements using four electrodes within each region: the contralateral-to-the-task (above the left hemisphere), prefrontal cortex (area of F3), contralateral sensorimotor cortex (area of C3), ipsilateral-to-the-task (above the right hemisphere) prefrontal cortex (area of F4), and the ipsilateral sensorimotor cortex (area of C4). The data are presented as mean values with standard deviations, as well as topographic maps of scalp potentials.

Subsequent analysis consisted of reconstruction of the regional sources’ locations of the ERP based on the 2-s-long averaged file but limited to the time of the ERP. BESA’s discrete multiple source analysis method (Scherg and Berg, 1996) was used separately for this purpose for each subject, task, and session. Details on selected features of this method that have the power to transform the EEG signal from the scalp back into the brain structures were described by Scherg et al. (2019). The number of sources was determined based on a principal component analysis (PCA) conducted using the BESA software of the data corresponding to the time when the residual value (unexplained by the dipole data) was less than 10% (Hoshiyama et al., 1997). According to Scherg (1992), PCA provides an *a priori* objective estimate of the lowest number of possible sources, which, together with the assumption of RV < 10%, allowed us to quantitatively analyze the number of sources between the subjects, sessions, and tasks. The source locations were estimated using a four-shell ellipsoidal head model. The location of each source was determined in the Talairach standard brain space (x, y, and z coordinates) and transformed to a Brodmann area (BA) in Talairach Daemon (Talairach Client 2.4.2), as presented in Tables.

## Results

### Vividness of Motor Imagery of Reaching for the Patient and Control

The vividness of the MIR for the patient was high (8.7, 8.4, 9.3, and 7.8) during the four measurement sessions from PRE to POST12. The vividness of the MIR for the control was high during the PRE, POST4, and POST12 sessions, but lower during the POST8 measurement session (9.4, 7, 5.5, and 9.2 for PRE, POST4, POST8, and POST12, respectively).

### ERP Analysis Related to Motor Imagery of Reaching for Patient

**Figure 2:**
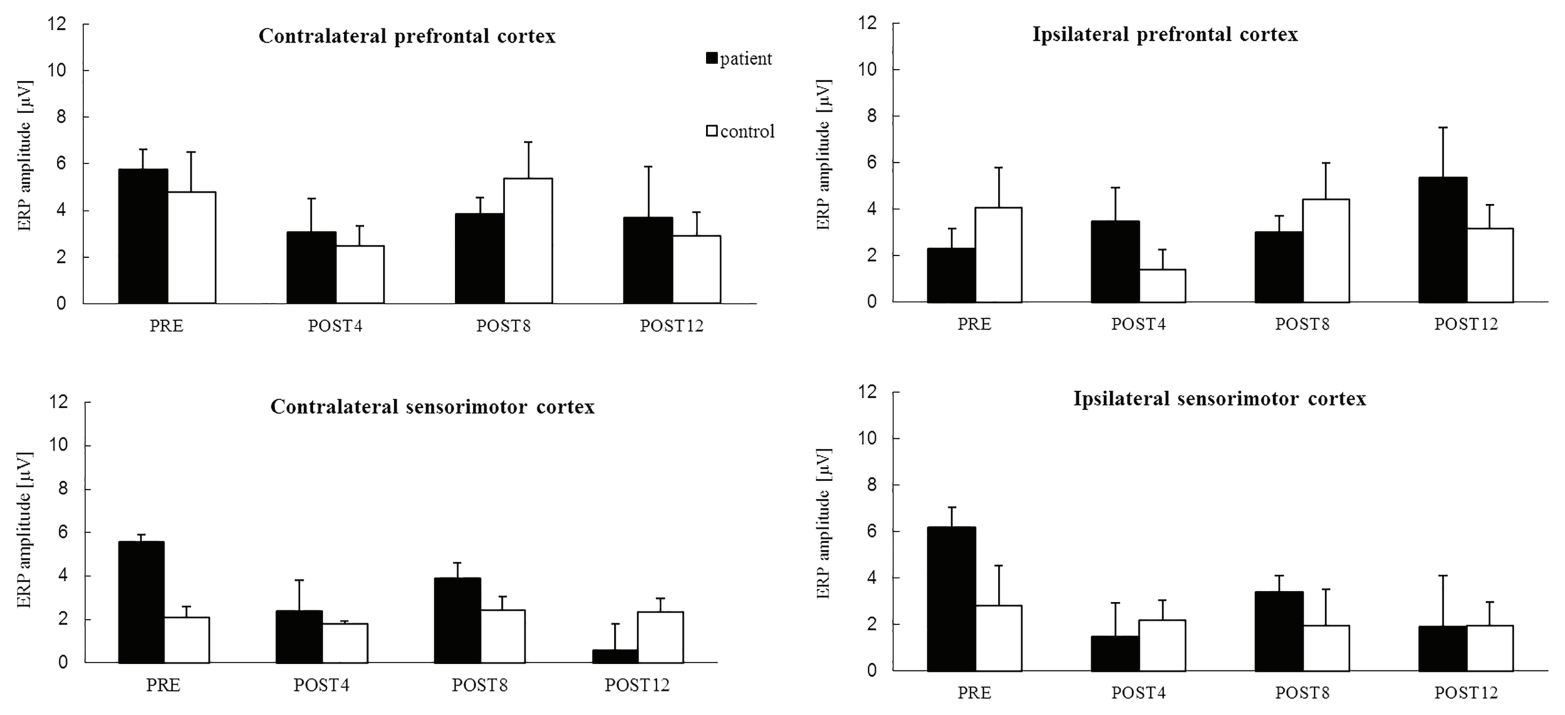
The mean value of ERP amplitude [*−1 (μV)] related to the motor imagery of reaching for the right upper limb from four channels in: the contralateral prefrontal cortex (above the **left** hemisphere) – area of standard “F3” channel, ipsilateral prefrontal cortex (above the **right** hemisphere) – area of standard “F4” channel, and contralateral sensorimotor cortex – area of standard “C3” channel and ipsilateral sensorimotor cortex – area of standard “C4” channel for the patient (black color) and control subject (white color) for sessions before 12 weeks of computer-aided imagery training (PRE) and after intervals of 4 weeks each (POST4, POST8, and POST12).

The results showed that in the contralateral prefrontal cortex and sensorimotor cortexes of both hemispheres, the ERP amplitude related to the MIR decreased with CAIT and increased only in the ipsilateral prefrontal cortex as a result of CAIT for the patient (Figure 2). The ERP amplitudes had relatively similar values in each of the four regions during the POST4 and POST8 sessions. Higher values above the prefrontal cortices of both hemispheres and lower values above the sensorimotor cortices above the right and left hemispheres were observed after completion of the training period (POST12; Figure 2).

### ERP Analysis Related to Motor Imagery of Reaching for Control

The mean values of the ERP amplitude in the contralateral‐ and ipsilateral-to-the-task sensorimotor cortexes were similar for all the measurement sessions for the control, i.e., they did not change noticeably over the training period (Figure 2). Unstable trends in the mean value of ERP amplitudes were observed in the prefrontal cortices of both hemispheres, with similarly high values observed during the PRE and POST8 sessions, but with low values observed during the POST4 session and after completion of the training period (POST12).

### Location of Regional ERP Sources Related to Motor Imagery of Reaching

**Figure 3:**
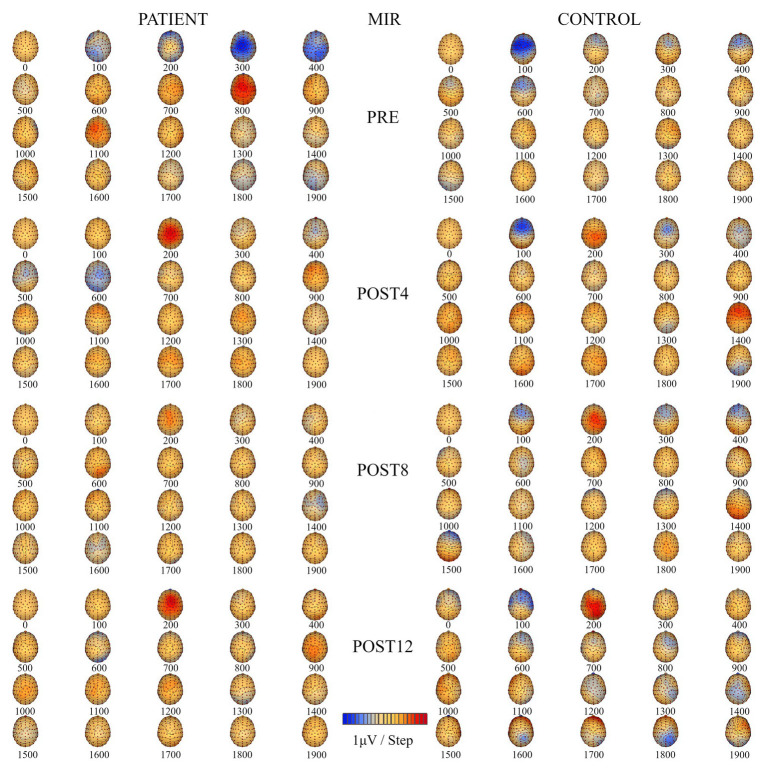
Topographic maps of electroencephalography (EEG) during motor imagery of reaching (MIR) of the right upper limb for the patient **(left)** and the control subject **(right)** for four measurement sessions (PRE, POST4, POST8, and POST12).

The results (Table 1) show that the number of ERP sources related to the MIR the right upper limb for the patient did not change over the course of the training. However, the location did change. The ERP sources for the patient related to the MIR task were located in the frontal lobe, the anterior cingulate cortex of the right hemisphere (BA 32), the middle temporal gyrus of the left hemisphere, and the occipital cortex of the right and left hemispheres (BA 18 and BA 19). After the full training period, the ERP sources were located in the dorsolateral prefrontal cortex of both hemispheres (BA 9, BA 10, and BA 46), the posterior cingulate cortex of the left hemisphere (BA 23), and the premotor cortex (BA 8) of the right hemisphere. The number of ERP sources related to MI reaching the control (Table 1) decreased over the course of training from 5 (PRE) to 3 (POST12). After the training period, the sources of the ERP of the MIR were localized in the premotor cortex of the left hemisphere (BA 8), the dorsolateral prefrontal cortex of the right hemisphere, and the posterior cingulate cortex of the right hemisphere (POST12). Topographic maps of EEG during MIR for the patient and the control are presented in Figure 3.

**Table 1:** Location (x, y, and z coordinates) of the regional sources of the ERP related to MIR of the patient (on the left side in the table) and control subject (on the right side in the table) expressed in lobe, Brodmann areas (BAs), or other from four measurement sessions (PRE, POST4, POST8, and POST12).

Patient	No.	x	y	z	Lobe/BAs/other	Control	No.	x	y	z	Lobe/BAs/other
MIR						MIR					
PRE	1	77.5	−10	−12	Frontal lobe	PRE	1	3	−22	26.6	23
	2	9.3	16.6	44.9	32		2	8.4	−79	−19	18
	3	−23	−98	−21	18		3	66.1	−29	0.5	22
	4	52.6	−90	4.7	19		4	44.7	54.3	33.5	9
	5	−68	−52	−4.2	21		5	−19	−4	50.8	6
POST4	1	24.7	22.9	−6.1	13	POST4	1	−0.2	−11	7	Thalamus
	2	19.1	−43	36.7	31		2	−47	56.2	21.7	10
	3	−47	−92	−20	Posterior lobe		3	52.6	39.9	26.8	46
	4	58.8	−70	−34	Posterior lobe		4	48.4	−49	−7.5	37
	5	−34	13.3	60.4	6		5	−61	23.9	19.1	45
POST8	1	5.7	−40	39.5	31	POST8	1	−47	48	23.6	46
	2	25.8	−61	7.2	30		2	53.5	44.6	−5.7	47
	3	−57	−26	58.1	Parietal lobe		3	3	−64	10.7	30
	4	−67	−67	3	37		4	59.1	40.9	28.8	46
	5	−57	48.6	21.6	46						
	6	73.5	−17	14.9	42						
POST12	1	−1.4	−59	14.4	23	POST12	1	2.6	−19	31	23
	2	7.2	18	50.4	8		2	−56	20	52.2	8
	3	48.3	58.8	21.6	10		3	41.6	66.1	18.3	10
	4	−60	27	40.9	9						
	5	64.9	32.7	24.5	46						

A negative x coordinate value indicates location in the left hemisphere, while a positive x coordinate value indicates location in the right hemisphere.

### Vividness of Motor Imagery of Grasping for the Patient and Control

The vividness of the MIG for the patient was 4.8, 6.2, 5, and 8 during the four measurement sessions from PRE to POST12. The vividness of the MIG was higher for the control (9.9, 8.2, 8.9, and 9.9) for the PRE, POST4, POST8, and POST12 sessions, respectively.

### ERP Analysis Related to Motor Imagery of Grasping for the Patient

**Figure 4:**
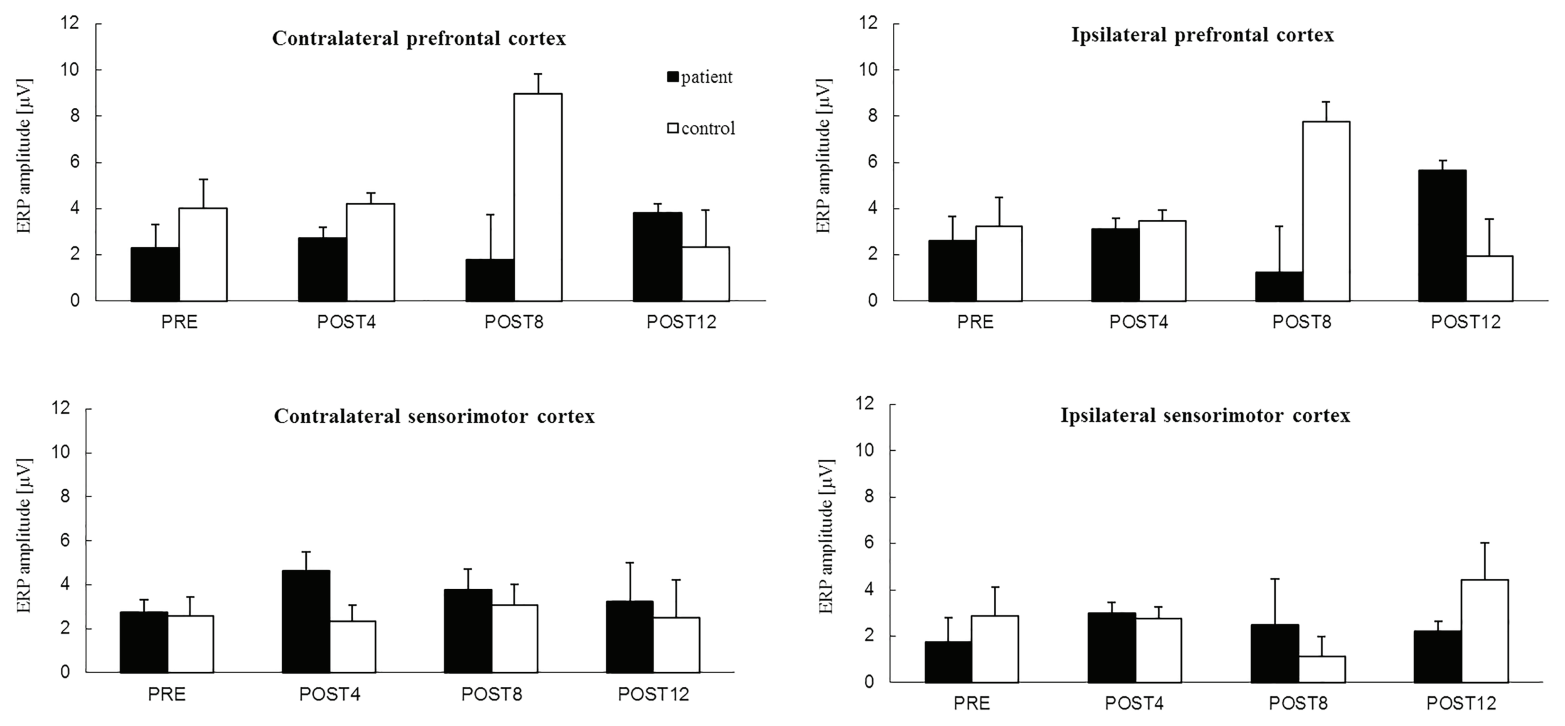
The mean value of ERP amplitude [*−1 (μV)] related to motor imagery of grasping of the right hand from four channels in: the contralateral prefrontal cortex (above **left** hemisphere) – area of standard “F3” channel and ipsilateral prefrontal cortex (above **right** hemisphere) – area of standard “F4” channel, contralateral sensorimotor cortex – area of standard “C3” channel and ipsilateral sensorimotor cortex – area of standard “C4” channel for the patient (black color) and the control subject (white color) for the session before 12 weeks of computer-aided imagery training (PRE) and after intervals of 4 weeks (POST4, POST8, and POST12).

The results show that the ERP amplitudes related to the MIG increased with CAIT for both the patient and control in each of the four regions (Figure 4) except for the ipsilateral prefrontal cortex for the POST8 measurement session. For the contralateral and ipsilateral prefrontal cortices, the mean values were approximately twice as high after the full CAIT period (POST12) before training. Increases in the ERP amplitudes for the sensorimotor cortexes of the right and left hemispheres were observed after 4 weeks of training (POST4) and remained higher in subsequent measurement sessions (POST8 and POST12) than those before training (PRE). During the POST4 and POST8 sessions, the ERP amplitudes were slightly higher for the sensorimotor cortexes above the right and left hemispheres than for the prefrontal cortices of both hemispheres.

### ERP Analysis Related to Motor Imagery of Grasping for Control

The mean values of the ERP amplitudes for the prefrontal and sensorimotor cortexes above the right and left hemispheres did not change after the first 4 weeks of training (from PRE to POST4) for the control (Figure 4). Similar values were observed for the prefrontal cortexes above the right and left hemispheres, while similar but slightly lower values were observed for the sensorimotor cortexes above the right and left hemispheres. Changes in the ERP amplitudes were observed after 8 weeks of training and were different for the prefrontal and sensorimotor cortices of both hemispheres. The ERP amplitudes of the prefrontal cortexes of both hemispheres increased greatly after 8 weeks of training (POST8), but decreased to lower values than before the training period after 12 weeks (POST12). The ERP amplitudes of the contralateral sensorimotor cortex remained similar throughout the training period (albeit with an increase noted at POST8) and doubled for the sensorimotor cortex above the right hemisphere by the end of the training period (POST12) in comparison to those in the PRE session.

### Location of Regional ERP Sources Related to Motor Imagery of Grasping

**Figure 5:**
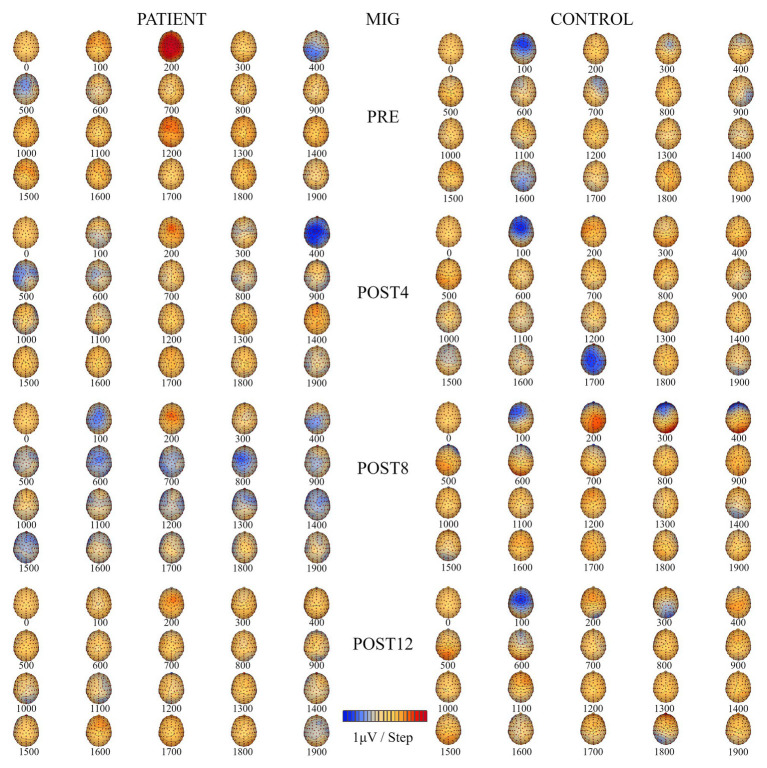
Topographic maps of EEG during motor imagery of grasping (MIG) of the right hand for the patient **(left)** and the control subject **(right)** for four measurement sessions (PRE, POST4, POST8, and POST12).

The number of ERP sources related to MI for grasping the right hand (Table 2) for the patient increased from four (PRE, POST4, and POST8) to five after the training. Before the training, the sources were located in the temporal lobe of the left hemisphere, the dorsolateral prefrontal cortex of the left hemisphere, and above the right hemisphere in the precuneus and occipital cortex. After the full training period, they were located in the premotor cortex, posterior lobe, occipital cortex of the left hemisphere, dorsolateral prefrontal cortex of the right hemisphere, and thalamus. For the control, the number of ERP sources related to the MIG task decreased after CAIT from six (PRE) to four (POST12) and were eventually located in the primary sensory cortex (BA 2) of the left hemisphere, dorsolateral prefrontal cortex, occipital cortex, and limbic lobe of the right hemisphere (Table 2). Topographic maps of EEG during MIG for the patient and the control are presented in Figure 5.

**Table 2:** Location (x, y, and z coordinates) of regional sources of the ERP related to MIG of the right side of the patient (on the left side in the table) and control subject (on the right side in the table), expressed in lobe or BAs from four measurement sessions (PRE, POST4, POST8, and POST12).

PATIENT	No.	x	y	z	Lobe/BAs/other	CONTROL	No.	x	y	z	Lobe/BAs/other
MIG						MIG					
PRE	1	−46	57.6	14.5	10	PRE	1	−3.4	−2.8	5.1	White matter
	2	31.7	35.6	33.5	9		2	38.8	−64	1.5	37
	3	3.1	−21	47.7	31		3	−36	−39	13.8	41
	4	−67	−66	−12	Temporal lobe		4	−42	48.1	40.3	9
							5	56.4	2.8	26.8	6
							6	−35	−74	57.9	Parietal lobe
POST4	1	−4.9	−21	25.8	23	POST4	1	7.2	−15	4.6	Thalamus
	2	61.8	39.4	23.4	46		2	−48	52.5	22.1	10
	3	65.2	−70	−11	19		3	52.1	43.7	29	46
	4	44.4	53.4	24.7	10		4	41.2	−72	−7.9	19
	5	−62	−77	3.8	37		5	−65	16.2	34.3	9
							6	−26	9.1	50.6	6
							7	48.2	−46	−11	37
POST8	1	28.5	−79	6.6	18	POST8	1	−2	33.7	−1.3	24
	2	−5.1	−22	41.6	31		2	−6.9	−67	26.9	31
	3	−35	1	−17	38		3	41.2	52.5	12.7	10
	4	59.2	43.2	24.8	46		4	−46	43.8	3	46
	5	−45	−98	5.1	19		5	−1.3	−8.9	43.9	24
POST12	1	11.8	−17	6.1	Thalamus	POST12	1	−61	−24	37.4	2
	2	−8.1	43.9	41.3	8		2	12.8	−66	3.5	18
	3	45.2	58.4	29.7	10		3	21.5	34.9	32.1	9
	4	−59	−61	−28	Posterior lobe		4	15.4	−13	32.8	Limbic lobe
	5	−39	−88	40.1	19						

The negative value of the x coordinate indicates location in the left hemisphere, while a positive value of the x coordinate indicates location in the right hemisphere.

## Discussion

MI is a cognitive process and a dynamic state during which the representation of a specific motor action is internally activated without any motor output (Jeannerod and Decety, 1995). In other words, MI is a part of motor action related to the planning of voluntary movements. They share overlapping structures of the brain, which explains why learning effects occur after MI training and why mental practice facilitates performance (Drickstein et al., 2004; Ranganathan et al., 2004; Allami et al., 2014; Avanzino et al., 2015; Sobierajewicz et al., 2016). In the present study, 12 weeks of training in the kinesthetic MI of a reaching-to-grasp task with a virtual environment (CAIT) was used for the first time in a congenitally amputated patient to evoke training-related changes in cortical activity as a result of neuroplasticity.

Our experiment shows that CAIT can be conducted in patients born without upper limbs with higher vividness of imagery for reaching than for grasping tasks. Our results confirm that CAIT can change the brain activation patterns in areas related to motor planning and the execution of reaching and grasping movements, and that the effect was more pronounced in the patient than in the control. These results show that CAIT has a different effect on the cortical activity related to the MI of a reaching task than on the cortical activity related to the MI of a grasping task, which could be partially related to the different sensorimotor and visuomotor transformations required for reaching and grasping tasks (Kandel et al., 2000). In this study, we chose to use mental training for a task involving reaching to grasp a book, because this type of movement is frequently used in daily life and is commonly used in healthy people. This gave us the opportunity to compare the effect of training on cortex activity between a congenitally amputated patient and a healthy control.

### Effects of Computer-Aided Imagery Training on Cortical Activity Related to the Motor Imagery of Reaching

The effects of 12 weeks of CAIT were observed in the contralateral (left) and ipsilateral (right) prefrontal and sensorimotor cortices in the patient but only in the prefrontal cortices of both hemispheres of the control. The ERP amplitudes of the sensorimotor cortices of both hemispheres in the patient decreased after the full training period and approached values similar to those of the control, for which they were stable throughout the whole training period (i.e., they did not change as a result of the training). Greater variability in ERP amplitudes over time was noted for the prefrontal cortices of both hemispheres for the patient and control. The ERP amplitudes for these areas, except for the ipsilateral prefrontal cortex, decreased after the entire training period.

The effects of training on the ERP source locations were different for the patient than for the control. In the control, we observed a reduction in the number of ERP sources located in the premotor cortex of the left hemisphere, dorsolateral prefrontal cortex, and posterior cingulate cortex of the right hemisphere. For the patient, the number of sources did not change, but the areas became more specific than those observed for the healthy control, namely: the premotor cortex of the right hemisphere, the dorsolateral prefrontal cortex of both hemispheres, and the left posterior cingulate cortex. These findings are consistent with those reported in several previous studies (Glover et al., 2012; Hétu et al., 2013; Pilgramm et al., 2016) in healthy subjects, indicating that planning a reaching task without a grasping part could be easier to achieve by mental training in people who were born without arms. This could be related to the fact that planning of this task as a part of goal-directed movement is based on visual information regarding the target location (Kandel et al., 2000). According to Sober and Sabes (2003), the first stage of planning of goal-directed reaching mainly relies on visual information, accounting for approximately 80% of the process with only 20% for proprioception. Additionally, planning of the reaching requires not only visual information about the target location, but also information about the position of the upper limb – especially the position of the proximal muscles – to prepare motor commands related to the direction of movement. Reilly and Sirigu (2011) found that changes in motor cortex representation of the upper limb in individuals born without arms depend on the level of the missing part of the limb, where more proximal parts are less changed. This could explain our results regarding the observed training-related changes in cortical activity related to the MIR. Additionally, Binkofski et al. (2001) showed that activity in various areas of the frontal cortex is related to motor imagery in the context of its trajectory, which corresponds to the MIR. This could explain why in our study, ERP sources were frequently located in the frontal cortex for each measurement session for both the patient and control (areas such as BA 6, BA 8, BA 9, BA 10, or BA 46). After the entire training period, most of the ERP sources were located in the frontal lobe (BA 8 and BA 10) for the control and four out of five ERP sources (BA 8, BA 9, BA 10, and BA 46) for the patient.

### Effects of Computer-Aided Imagery Training on Cortical Activity Related to Motor Imagery of Grasping

The training increased the ERP amplitudes related to the MIG for the prefrontal and sensorimotor cortices of the right and left hemispheres in the patient. The number of ERP sources also increased from four to five as a result of the training, and were located in the premotor cortex, occipital cortex, posterior lobe of the left hemisphere, right dorsolateral prefrontal cortex, and thalamus. The localization of ERP sources corresponded to the areas noted by Glover et al. (2012). The authors identified large networks of brain structures related to the online control of the execution of goal-directed movement. This included the sensorimotor cortex, cerebellum, supramarginal gyrus, and superior parietal lobule. In our study, after the entire training period, the ERP source was localized in the sensory cortex (BA 2) only in the control. We found that the effects of training were smaller for the control. It is well known that the parameters of an object that should be grasped (such as weight and size) are essential in the planning phase of grasping as a part of goal-directed movement (Kandel et al., 2000). This could explain why ERP sources were often localized in the visual cortex (BA 17, BA 18, and BA 19) in both the patient and control. The ERP amplitudes for the prefrontal cortices of both hemispheres and the contralateral sensorimotor cortex remained stable (except for higher values during POST8), while the ERP amplitude of the ipsilateral sensorimotor cortex increased over the full course of the period for the control. After the training (POST12), the ERP sources were located in the primary sensory cortex of the left hemisphere, dorsolateral prefrontal cortex, and occipital cortex of the right hemisphere in the control. The different effects of CAIT on the sensorimotor cortices of both hemispheres (the increase in the patient and decrease in the control) could be related to the inhibition of movement that occurs during MI in healthy people (Solodkin et al., 2004; Kasess et al., 2008), which may not be the case in a congenitally amputated patient.

### Comparison of the Effects of Computer-Aided Imagery Training on Cortical Activity Related to Different Tasks (Motor Imagery of Reaching vs. Motor Imagery of Grasping)

Our results show that training had a smaller effect on the MIR (with a high level of vividness of imagery before the training that remained stable during the training period) and larger effects on the MIG (where the vividness of imagery was increased with training) in the patient. These findings can be compared to those of Williams et al. (2002), who reported that the vividness of imagery is associated with the level of neural activation in motor and related areas. These results could also be related to a greater familiarity with the reaching task for the patient who used to perform this type of action with her lower limbs. The MIG is a much more difficult task. However, with the support of applied training with virtual sensory feedback, the cortical activity related to this task increased. Our results also show that CAIT had different effects on the activity of the sensorimotor cortices of both hemispheres – as expressed in terms of the ERP amplitudes related to the MIR – compared to those for the MIG. The activity of the sensorimotor cortices of both hemispheres decreased during MIR as a result of training and increased during MIG. The ERP amplitudes of the sensorimotor cortices of the right and left hemispheres in the control were stable except for an increase in the right sensorimotor cortex after the full training period. The training increased ERP amplitudes in the prefrontal cortices of both hemispheres and for both tasks in the patient (except for the contralateral prefrontal cortex during MIG after 12 weeks of training). The effects of CAIT on the ERP amplitudes in the control were observed only for the prefrontal cortices of the right and left hemispheres and were different for the MIR task (unstable trends) than for the MIG task (increased during the POST8 session and then decreased by half after the full training period). Similarly, Allami et al. observed greater effects of MI training on frontal lobe activity, especially over the premotor cortex. CAIT also had different effects on ERP sources related to MIR and MIG tasks. The number of sources did not change for the patient during the MIR task, but the locations changed to more specific ones – similar to those for the healthy control. For the MIG task, an increase in ERP amplitudes was accompanied by an increase in the number of sources. Our results are consistent with those of an fMRI study of reaching and grasping tasks, in which Glover et al. (2012) reported activity in different brain areas related to pre-movement planning of reaching and online control that was more specific to the neural control of grasping tasks. Their results showed that these two tasks could involve different sub-processes of planning, which could explain, in part, why CAIT had different effects on those tasks in our study.

In our study, smaller effects of CAIT were observed on the ERP amplitude in the control, but the number of sources was reduced for the MIR and MIG tasks. This could be a result of the optimization of cortical activity related to the tasks after CAIT in the control.

## Conclusion

Although our study obtained results from only one patient and one control subject, our results confirm that 12 weeks of CAIT on a reaching-to-grasp task produced more notable cortical changes in a patient with congenital bilateral transverse upper-limb deficiency than in a healthy control subject. For the patient, training had smaller effects on the MIR and greater effects on the MIG. The larger number of ERP amplitude sources for the MIR and MIG tasks in the congenitally amputated patient indicates that the MIR and grasping at the cortical level was modulated by a higher number of areas of the brain in the patient than in the control. The changes observed in the activation patterns indicate CAIT-induced neuroplasticity.

Despite the study’s novel findings, there are certain limitations that are mainly related to the case study’s characteristics, such as issues of reliability, validity, and the problem of determining the real causes of the described effects. The major limitation of our study is that our results were obtained from only one patient and one healthy control subject, which prevents generalization of the results.

Nevertheless, this study presents an unusual situation of using mental training that can be used in patients with congenital limb deficiency. This has never been reported before and opens up the possibility of upper-limb transplantation in such patients in the future if the effects of training-induced neuroplasticity would be confirmed by studies with larger sample sizes.

## Data Availability Statement

The raw data supporting the conclusion of this article will be made available by Principal Investigator K. Kisiel-Sajewicz (grant number DEC-2011/03/B/NZ7/00588) on reasonable request.

## Ethics Statement

The studies involving human participants were reviewed and approved by Ethical Committee of the University School of Physical Education in Wrocław, Poland. The patients/participants provided their written informed consent to participate in this study.

## Author Contributions

KK-S, AnJ, JMa, ArJ, MK, and AW contributed conception and design of the study. JMe and KK-S wrote the first draft of the manuscript. ŁK collected EEG data under supervision of KK-S. ŁK conducted training. All authors contributed to manuscript revision, read, and approved the submitted version.

### Conflict of Interest

The authors declare that the research was conducted in the absence of any commercial or financial relationships that could be construed as a potential conflict of interest.
